# Haemodynamics of stent-mounted neural interfaces in tapered and deformed blood vessels

**DOI:** 10.1038/s41598-024-57460-w

**Published:** 2024-03-27

**Authors:** Weijie Qi, Andrew Ooi, David B. Grayden, Nicholas L. Opie, Sam E. John

**Affiliations:** 1 Department of Biomedical Engineering, The University of Melbourne, Parkville, Australia; 2 Department of Mechanical Engineering, The University of Melbourne, Parkville, Australia; 3 Graeme Clark Institute, The University of Melbourne, Parkville, Australia; 4 Vascular Bionics Laboratory, Department of Medicine, The University of Melbourne, Melbourne, VIC Australia; 5 Florey Institute of Neuroscience and Mental Health, Melbourne, Australia

**Keywords:** Biomedical engineering, Mechanical engineering

## Abstract

The endovascular neural interface provides an appealing minimally invasive alternative to invasive brain electrodes for recording and stimulation. However, stents placed in blood vessels have long been known to affect blood flow (haemodynamics) and lead to neointimal growth within the blood vessel. Both the stent elements (struts and electrodes) and blood vessel wall geometries can affect the mechanical environment on the blood vessel wall, which could lead to unfavourable vascular remodelling after stent placement. With increasing applications of stents and stent-like neural interfaces in venous blood vessels in the brain, it is necessary to understand how stents affect blood flow and tissue growth in veins. We explored the haemodynamics of a stent-mounted neural interface in a blood vessel model. Results indicated that blood vessel deformation and tapering caused a substantial change to the lumen geometry and the haemodynamics. The neointimal proliferation was evaluated in sheep implanted with an endovascular neural interface. Analysis showed a negative correlation with the mean Wall Shear Stress pattern. The results presented here indicate that the optimal stent oversizing ratio must be considered to minimise the haemodynamic impact of stenting.

## Introduction

Venous sinus stenting is a conventional intervention that aims to improve hypertension-related narrowing of blood vessels within the cranial cavity. Elevated intracranial pressure exerts substantial amounts of compression on the cerebral venous system, leading to constriction of venous sinuses, including the superior sagittal sinus (SSS), transverse sinus (TS), or sigmoid sinus (SS), resulting in symptoms such as headaches and tinnitus^1^. The deployment of one or multiple venous stents serves to restore blood flow and alleviate these symptoms. More recently, venous sinus stenting has gained renewed attention with the emergence of stent-electrode devices. While traditionally stenting is applied to treat stenosis, the stent-electrode array in this study functions as a neural implant. These implants are currently being considered as brain computer interfaces and for recording from and stimulating the brain and peripheral nervous system^2^. The device records brain signals of paralysed patients within healthy venous sinuses near the active brain regions^3,4^. The utilization of venous sinus stents as conduits is increasingly favoured due to their minimally invasive nature and reduced risk compared to conventional implanted electrode arrays that have direct contact with brain tissue. With the growing interest in venous sinus stenting, there is an increasing need to understand venous stent complications, particularly neointimal hyperplasia, a facet currently absent in the literature and clinical data.

Neointimal Hyperplasia is characterised by excessive neointimal tissue growth on the innermost layer of the blood vessel wall after vascular interventions which can occur following stent implantation^5^. Hyperplasia is the major cause of in-stent stenosis, characterised by a severe narrowing (greater than 50%) of the blood vessel lumen after stenting, occurring in approximately 10% of patients receiving a stent^6^. With the rapid expansion of stent technologies, including neural interfaces using a stent scaffold, there is a compelling need to better understand venous neointimal hyperplasia within implanted blood vessels.

Haemodynamics (blood flow) plays an essential role in the development of neointimal hyperplasia^7,8^. Blood flow exerts mechanical stimuli, including pressure force and Wall Shear Stress (WSS), to the inner vessel wall covered by a thin layer of endothelial cells. The WSS is sensed by the endothelial cells that, in turn, react to variations in shear conditions^7^. A disturbance of the mechanical environment can transfer cells from an inactive (quiescent) to an active (pro-inflammatory) state, initiating and accelerating endothelial growth^8^. A low shear environment (WSS < 0.5 Pa) can induce cell proliferation, while a high wall shear stress gradient (WSSG > 200 Pa/m) can induce cell accumulation downstream^9^. When a stent is introduced into the blood vessel, the presence of stent struts will abruptly modify the blood flow pattern and the shear stress. There is a potential that a stent will elevate the risks of neointimal overgrowth after implantation. Therefore, studying haemodynamics in stented blood vessels is crucial to predict the risk of neointimal hyperplasia to enhance stent design and safety.

To study blood flow dynamics, extensive research^8–10^ has been conducted using Computational Fluid Dynamics (CFD). Studies have revealed that stents created stagnant or circulated flow patterns characterised by low shear stress on nearby blood vessel walls, where greater amounts of neointimal thickening were observed^11^. However, many CFD studies have assumed a cylindrical blood vessel lumen with minimal vessel wall deformation and tapering after stent implantation, which was found to provide inaccurate results^11,12^. Studies that have used deformed models found that the stent deployment ratio (stent-to-artery ratio) had a great influence on the blood flow in stented arteries, although the optimal stent deployment ratio and haemodynamics have not yet been well quantified, especially for tapered blood vessels^13^.

Compared to the arterial stent literature, there have been fewer studies on venous sinus stents. Neointimal growth in venous stenting has not been previously reported in clinical studies with supporting patient data. However, in recent years, Opie et al.^14^ identified neointimal growth in sheep Superior Sagittal Sinus (SSS) over 190 days after implantation of a neural interface based on a stent-mounted electrode array with a growth pattern that varied along the length of the stented segment. Furthermore, factors that may contribute to the variable tissue growth in the venous sinus were not quantified in the previous work. Therefore, there is a need to better understand tissue growth in stented venous sinus due to growing interest in using endovascular stents in the cerebral venous sinus^15^.

In the present study, we used CFD to evaluate the haemodynamic impacts of a stent and an endovascular neural interface on idealised human and sheep venous sinus models. The haemodynamic impacts of deformation and blood vessel tapering were quantified using various stent-to-vein ratios for the stented blood vessel. Simulation results were compared with tissue growth data from sheep. The current work sheds light on neointimal growth after stenting in venous blood vessels in the brain. It takes the first steps towards realising customisable endovascular neural interfaces and stents to minimise vascular remodelling and the degree of blood vessel narrowing after implantation.

## Methods

### Geometry constructions

**Figure 1:**
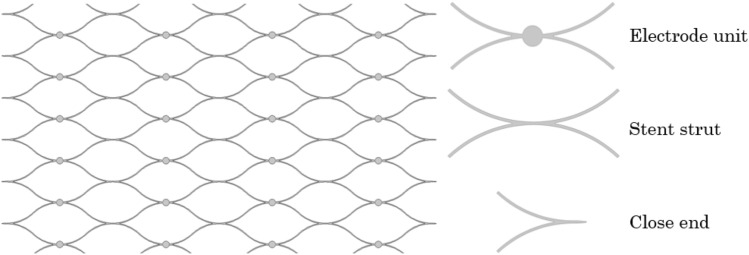
The design pattern of the stent-based neural interface. The stent had a rectangular strut profile (70 μm width and 50 μm thickness). Twenty-four electrodes (D = 500 μm) were attached to the stent struts.

All human venous sinus and stent-based neural interface models were generated using the design module of COMSOL Multiphysics (v. 5.6, Stockholm, Sweden)^16^. The stent model was composed of electrodes mounted on a nitinol scaffold where disc electrodes were fused, as depicted in Fig. 1. The stent was then placed inside an idealised human venous sinus, which was represented as either a hollowed cylinder or a hollowed cone with parameters from the Superior Sagittal Sinus^17,18^. Hollowed cones were used to represent the tapering feature of the blood vessel, which was the ratio between the diameters of the flow inlet and outlet. Five numerical cases were included in this study. Cylindrical blood vessels were constructed for Cases 1, 2, and 3, whereas conical blood vessels with various tapering ratios (b = 1:1.1 and 1:1.2) were used in Cases 4 and 5 (as summarised in Table 1).

**Table 1:** Summary of numerical cases with stent deployment and blood vessel tapering ratios.

Case no	Features	Stent deployment ratio (a)	Vessel tapering ratio (b)
1	Control	1	1:1
2	Slightly deformed	1.05	1:1
3	Deformed	1.1	1:1
4	Slightly tapered	1.1	1:1.1
5	Tapered	1.1	1:1.2
6	Sheep-specific	–	–
7	Sheep-specific	–	–
8	Sheep-specific	–	–

In addition to these computational models, three idealised sheep models (Cases 6–8) were constructed as curved cones with the curvature and diameters obtained from the CT scans^19^. The shape of the original sheep blood vessels could not be obtained from the CT scans due to uneven tissue growth and strong metal artefact. The geometries of the three stent-based neural interfaces were successfully segmented from CT scans using 3D Slicer^20^. The 3D geometries were then smoothed with Autodesk Meshmixer (Autodesk Inc., San Rafael, CA, USA).

### Finite element analysis (FEA) and computational fluid dynamics (CFD) simulations

**Figure 2:**
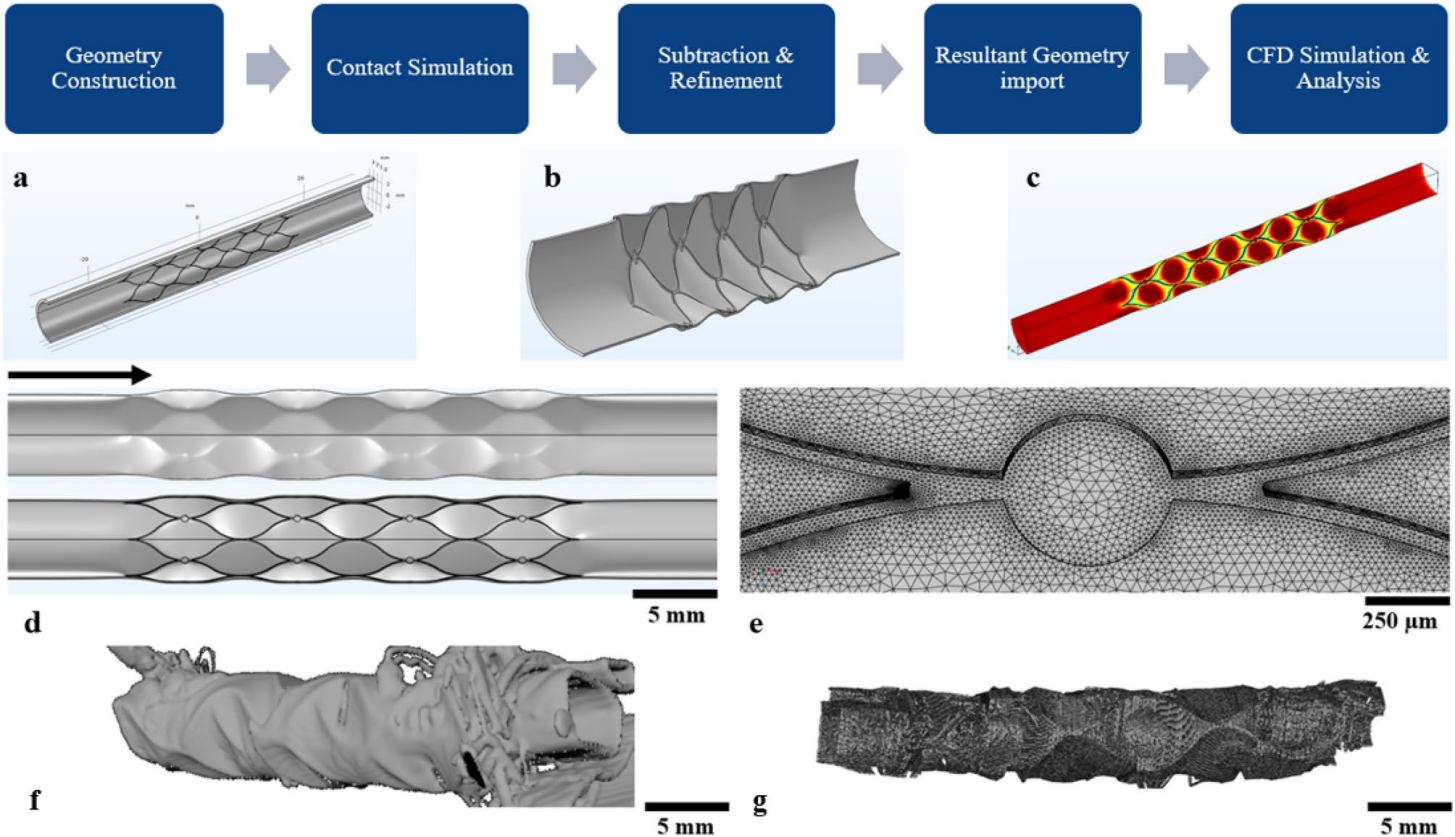
The workflow for deformed blood vessel wall model generation and CFD analysis. (**a**) A generalised blood vessel model with a stent-like neural interface. (**b**) The blood vessel was deformed by the expansion of the stent struts after the mechanical simulation. (**c**) Blood flow was simulated on the new deformed geometry to produce WSS results for the deformed model. (**d**) The deformed geometry is viewed from the outside and inside. (**e**) Tetrahedral mesh of the stent and blood vessel wall. (**f**) Blood vessel 3D segmentation from Micro-CT slices for sheep 2. (**g**) Blood vessel 3D coordinates extracted from Micro-CT slices for sheep 2. Stent artefacts were present, which made the reconstruction non-ideal for simulation.

The structural mechanics module of COMSOL Multiphysics (v. 5.6, Stockholm, Sweden) was used to simulate stent deployment in the venous sinus wall. A non-linear contact problem was solved between the self-expanding neurovascular stent-based neural interface and the venous sinus wall (Fig. 2a), where the expansion of the stent was defined by a prescribed radial displacement on the stent strut. The stent diameter increased evenly over time along the length of the stent in the mechanical simulation. Both the inlet and outlet of the blood vessel were stationary during stent expansion. Friction and stent movements in the longitudinal direction were ignored. Material properties of venous sinus and nitinol stent were from the literature (Table 2)^21,22^. Mechanical simulations were run on the Spartan supercomputer facility at the University of Melbourne. The deformed geometries (Fig. 2b) were generated from the displacement results and exported for blood flow simulation (Fig. 2c) at different deployment ratios (a = 1.0, 1.05, and 1.1), which was the ratio between lumen diameter after stenting to healthy lumen diameter. All stent-to-vein ratios were within the range in the literature^11,12^. Symmetry was used to reduce the computational cost and results were verified with a full model.

**Table 2:** Material properties of the venous sinus wall and nitinol stent for the mechanical simulation.

Name	Elastic modulus	Poisson’s ratio	Shear modulus	Mass density	Tensile strength	Yield strength
Sinus wall	30.69E6 (Pa)	0.49	4.66E5 (Pa)	1102 (kg/m^3^)	4.9E6 (Pa)	4.13E6 (Pa)
Nitinol	8E10 (Pa)	0.33	1.08E10 (Pa)	6450 (kg/m^3^)	9E8 (Pa)	1E8 (Pa)

The deformed geometries (Fig. 2d) were processed with Boolean subtraction to generate the flow domain for the stented blood vessel (Fig. 2e), which was subsequently meshed with tetrahedral elements with a minimum element size of 0.01 mm and with four boundary layers. Using the CFD module in COMSOL Multiphysics, a laminar flow was applied for the venous flow. The blood had a density of 1060 kg/m^3^ and shear-thinning viscosity using the non-Newtonian Carreau model (μ_0_ = 0.056 Pa s, μ_∞_ = 0.0035 Pa s, λ = 3.313 s, n = 0.3568)^23^. A fully developed flow profile was applied at the inlet of the blood vessel with a flow rate of 285 ml/min^24^. For the sheep blood vessel models, a different flow rate of 53 ml/min was applied based on the ultrasonic Doppler method^25^. Mesh convergence verified that the simulated WSS results did not vary with the mesh size of the model. The tangential WSS was computed using

1 $$\tau_{w} = {\varvec{\tau}}_{{\varvec{t}}} - \left( {{\varvec{\tau}}_{{\varvec{t}}} \cdot {\varvec{n}}} \right)\user2{n,}$$

where ${\varvec{\tau}}_{{\varvec{t}}}$ is the wall traction vector calculated from the stress tensor and ${\varvec{n}}$ is the surface normal. WSS ($\tau_{w}$) below 0.5 Pa was defined as low WSS^9,11^.

The Wall Shear Stress Gradient (WSSG) is the magnitude of the spatially varying wall shear stress and was defined as

2 $$WSSG = \sqrt {\left( {\frac{{\partial \tau_{w} }}{\partial x}} \right)^{2} + \left( {\frac{{\partial \tau_{w} }}{\partial y}} \right)^{2} + \left( {\frac{{\partial \tau_{w} }}{\partial z}} \right)^{2} } .$$

The WSSG measured the spatial change of WSS along the blood vessel, where a high WSSG indicated a rapid change of the WSS. WSSG > 200 Pa/m was defined as high WSSG^9,26^. In our model, the gradient in the *x* direction (major blood flow direction) contributed the most to the WSSG.

### Data analysis

Tissue growth data were obtained from a previous animal study using high-resolution micro-CT imaging^14^, where animals were implanted with the stent-electrode interface (Fig. 2f). Stent-associated tissue thickness was measured from CT and histological slices (Fig. 2g). Tissue thickness was averaged over the circumference of the blood vessel and computed along the length of the blood vessel. In the CFD model, the WSS results were exported to MATLAB to compute the circumferential averages and compare them with the average tissue growth.

## Results

### Wall shear stress (WSS)

**Figure 3:**
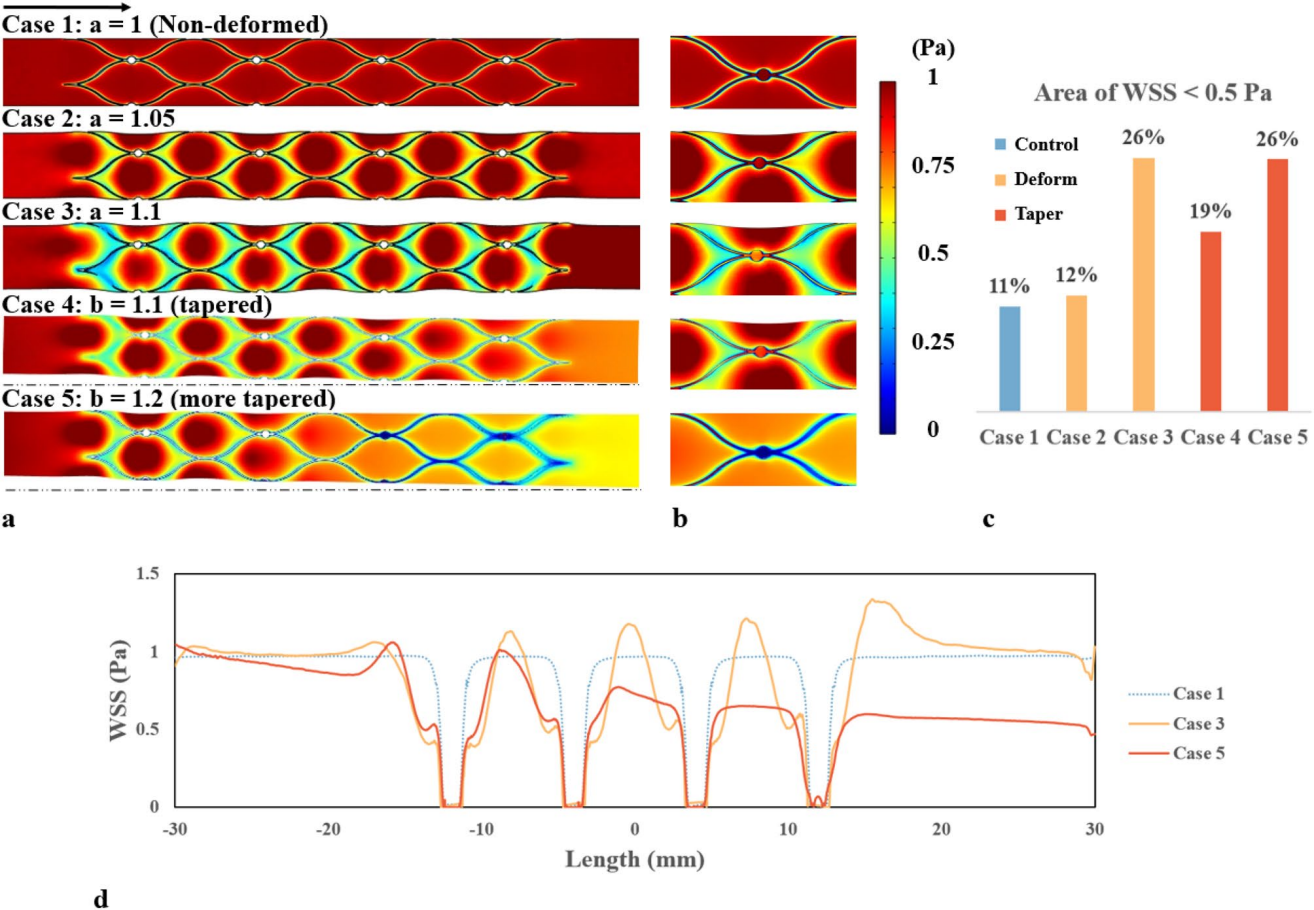
CFD results for wall shear stress (WSS). (**a**) WSS contour of the blood vessel wall (the black arrow indicates the blood flow direction). (**b**) A magnified view of the WSS pattern around the stent and electrode. (**c**) Histogram showing the area of WSS < 0.5 Pa with various stent-to-vein ratios. The area of the stent is not included in the area percentage. (**d**) Axial WSS distribution along the length of the blood vessels.

Figure 3 showed the WSS distribution of the stented venous wall. In Case 1 (no deformation), the WSS was reduced in regions immediately surrounding the edges of the stent strut and the electrodes. The area of low WSS was 11% of the total stented area (Fig. 3c). In Cases 2 (deformation ratio 1.05) and 3 (deformation ratio 1.1), the reduction of WSS became increasingly prominent with the increase in deployment ratio. A wider spread could be observed in both cases. The area subject to a low WSS rose to 12% and 26% (Fig. 3c). In Cases 4 (deformation ratio 1.1, taper ratio 1.1) and 5 (deformation ratio 1.1, taper ratio 1.2), the tapering feature of the blood vessel caused a gradual decrease of WSS along the vessel length. In Case 4, the low WSS region around the stent was more obvious at the proximal (tapered) end of the stent than at the distal end. The area of low WSS caused by the stent was 19%. In Case 5, there was an abrupt decrease of the WSS at the distal end due to poor contact between the stent and the blood vessel wall. The area of low WSS for Case 5 increased to 26% (Fig. 3c). In addition, the WSS was plotted along the axial line of the blood vessels. Cases 3 (with deformation) and 5 (with deformation and tapering) resulted in a more variable WSS along the length of venous sinuses compared to Case 1.

### Wall shear stress gradient (WSSG)

**Figure 4:**
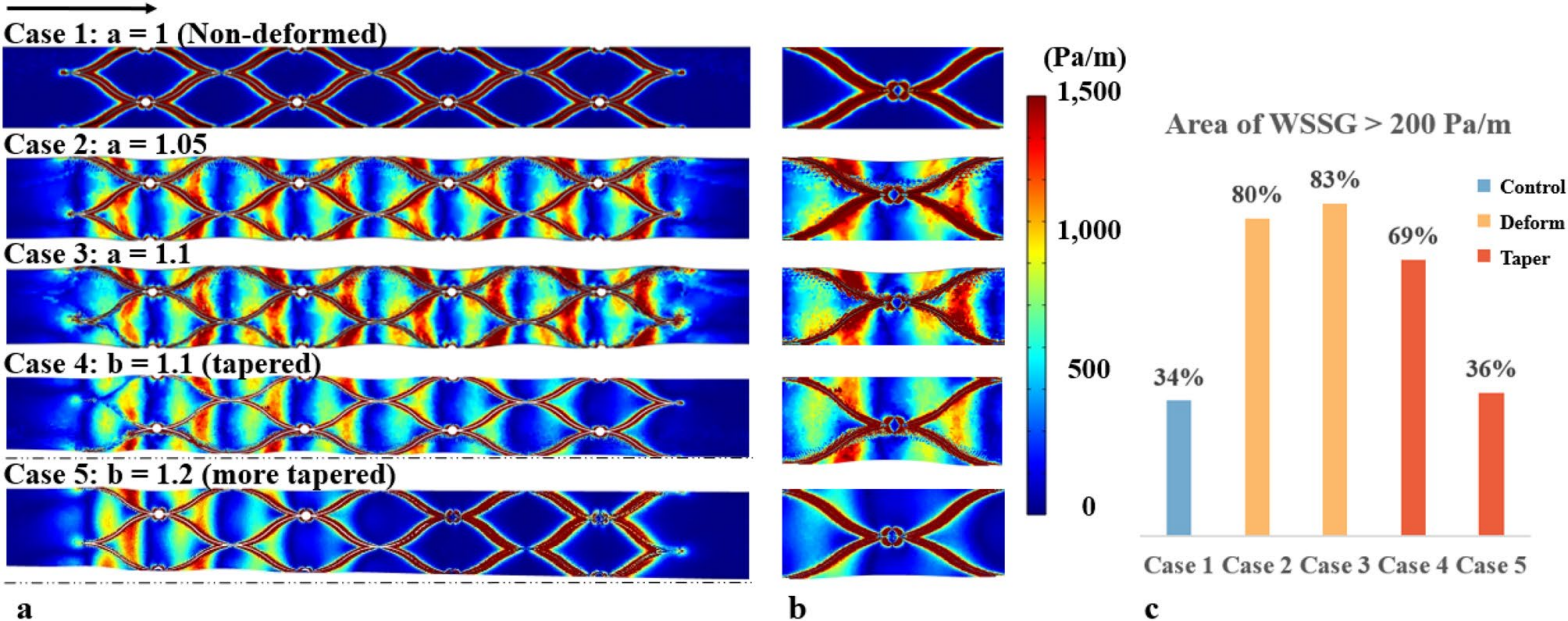
CFD results for wall shear stress gradient (WSSG). (**a**) WSSG contour of the venous wall (the black arrow indicates the blood flow direction). (**b**) A magnified view of the WSSG pattern around the stent and electrode. (**c**) Histogram showing Area of WSSG > 200 Pa/m with various stent-to-vein ratios. The area of the stent is not included in the area percentage.

Figure 4 depicts the WSSG and the area subject to a high WSSG (> 200 Pa/m). In Case 1 (no taper non-deformed), a high WSSG was observed around the stent struts and electrodes (Fig. 4b), indicating that the WSS varied rapidly (with an order of 1000 Pa/m) along the blood flow direction. The area of high WSSG contributed to 34% of the total area of the stented vessel (Fig. 4c). In Case 2 (deformation ratio 1.05) and Case 3 (deformation ratio 1.1), a WSSG between 500 and 1000 Pa/m was observed in the deformed venous wall region (Fig. 4a). The area of high WSSG histogram shows that deformation (Cases 2 and 3) increased the area of high WSSG by nearly 50% compared to the non-deformed idealised model (Case 1). In Cases 4 (taper ratio 1.1) and 5 (taper ratio 1.2), the proximal end of the stented region had a similar WSSG pattern due to deformation. However, high WSSG was mainly found around the stent and the area reduced to 69% and 36% in Cases 4 and 5 respectively (Fig. 4c).

### Flow streamlines

**Figure 5:**
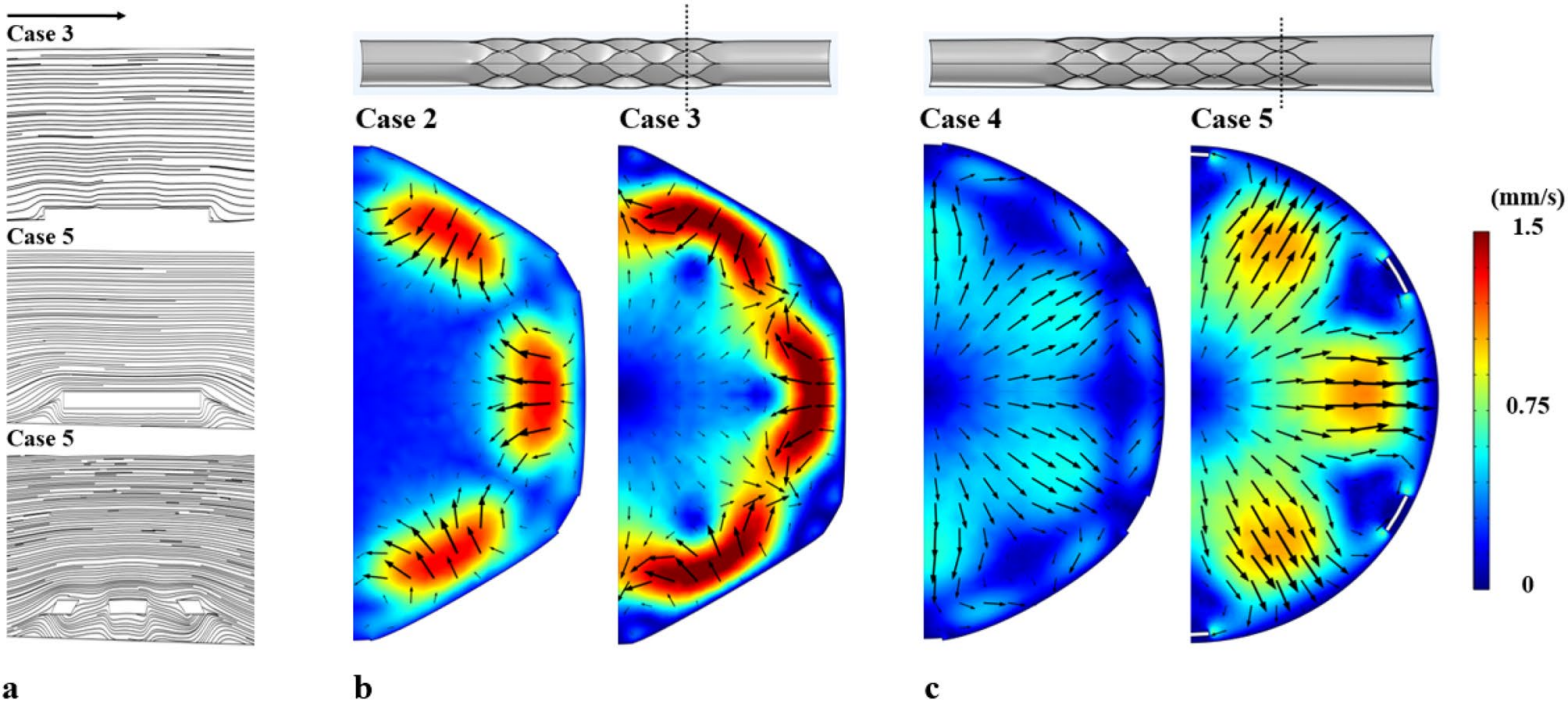
(**a**) Streamlines of blood flow over the stent struts and electrodes for Case 3 (deformation) and Case 5 (tapering). (**b**) Secondary flow magnitude at the blood vessel cross-section (dotted line) for Cases 2 and 3 without tapering. (**c**) Secondary flow magnitude at the blood vessel cross-section for Cases 4 and 5 with tapering.

Figure 5a illustrates the flow streamlines near the stent strut and electrode at the distal end of the stent. In Case 3 (deformation ratio 1.1), the stent was well apposed on the blood vessel wall, where a smoothed streamline was observed over the strut. In contrast, in Case 5 (taper ratio 1.2), flow disturbance characterised by twisted streamlines could be observed around the corners of the malposed struts. Secondary flow contours in Fig. 5b, c implied that stent deployment altered the cross sections of the blood vessel lumen. In Case 2 (deformation ratio 1.05) and Case 3 (deformation ratio 1.1), deformation induced secondary flow (1–1.5 mm/s) from the straightened venous wall towards the centre. The magnitude increased dramatically with an increase in the deployment ratio. In Case 4 (taper ratio 1.1) and Case 5 (taper ratio 1.2), tapering caused a secondary flow pattern away from the centre. The magnitude increased slightly from 0.5 to 1 mm/s with an increase in the tapering ratio.

### Mean wall shear stress and tissue growth thickness

**Figure 6:**
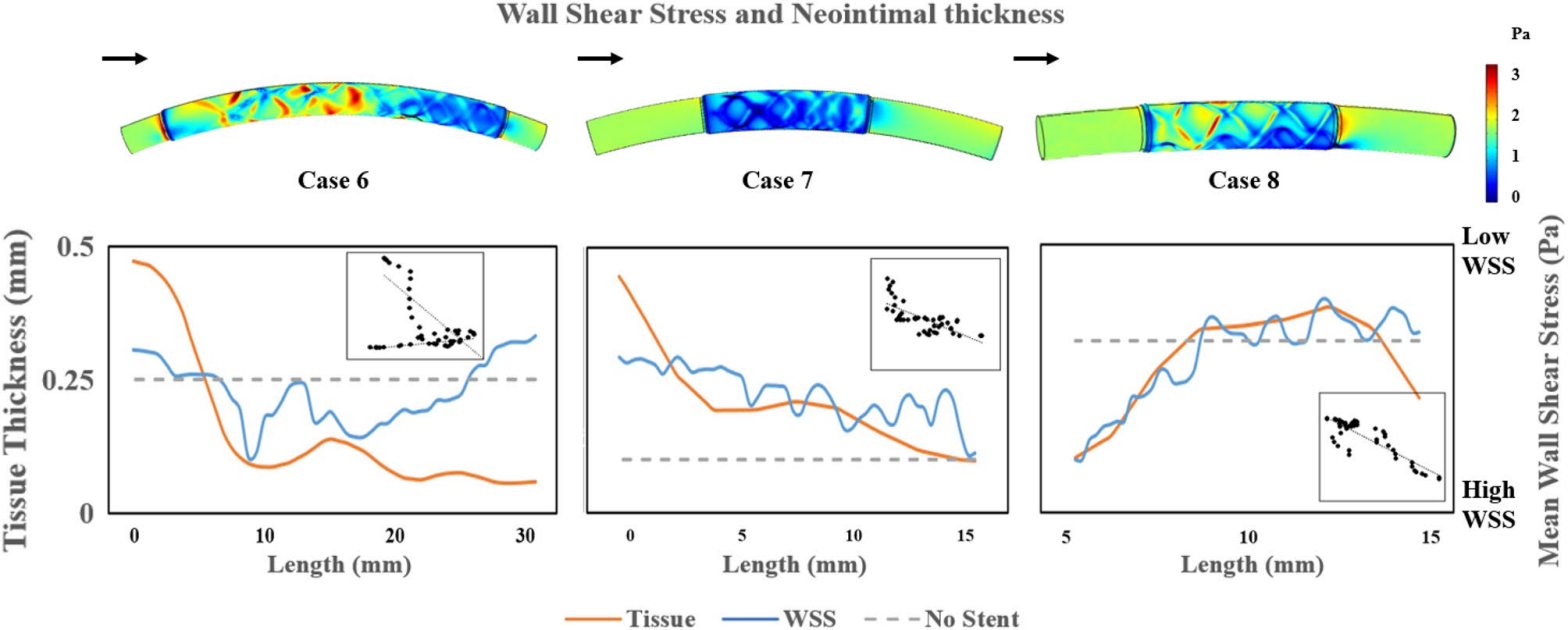
Comparison to Experimental Results. Top—WSS contour of the stented sheep blood vessel. Bottom—Blue: Circumferential average WSS along the length of the simulated blood vessel, computed from the WSS contour above; the WSS axis was flipped to compare trends between WSS and tissue thickness. Orange: Stent-associated tissue thickness in three sheep measured using Micro-CT imaging. Dashed line: Mean WSS value in the sheep blood vessels without a stent inside. Inset: Correlation between WSS and tissue thickness.

Figure 6 shows the WSS contour and comparisons of mean WSS with the average tissue thickness in three implanted sheep (Cases 6–8). Regions subject to WSS less than 0.5 Pa were primarily found around stent struts for all cases and the flow downstream for Cases 6 and 8. Regions subject to WSS greater than 2 Pa were observed near the outer bend of Case 6 due to longer vessel curvature than Cases 7 and 8. The three mean WSS plots in Fig. 6 (blue lines) show distinctive and non-uniform patterns in the stented region, varying between 0 and 3 Pa. In the sheep tissue thickness data (orange line), the tissue thickness at the proximal end was greater than the distal end for Cases 6 and 7. The peak of tissue thickness corresponded roughly to the regions with low WSS and vice versa. All the correlation plots showed moderate to strong levels of negative correlation between the mean WSS and tissue thickness, except for Case 6 with the length between 15 and 30 mm (Table 3).

**Table 3:** Correlation coefficient and *p* values for Cases 6–8.

Case no	Pearson’s correlation coefficient (r)	*p* value
6 (0–15 mm)	− 0.80	< 0.001
6 (15–30 mm)	0.73	< 0.001
7	− 0.72	< 0.001
8	− 0.86	< 0.001

## Discussion

We are the first to evaluate the haemodynamics in a cerebral blood vessel stented with an endovascular neural interface in idealised human and sheep Superior Sagittal Sinus models. The venous stenting literature lacks computational simulations that address venous blood flow and venous neointimal tissue growth in venous sinus stents. The interaction between venous stents and the venous blood vessel wall, as well as the factors influencing venous hemodynamics, remains unclear. Veins differ from arteries in terms of anatomical structures, blood flow characteristics, and optimal stent designs. Our study suggests that venous wall deformation may be a major contributor to changes in venous blood flow. This highlights the importance of considering venous deformation in mechanical simulations, which can provide more accurate estimates than computational fluid dynamics (CFD) analyses of rigid cylindrical blood vessels. As expected, the blood flow simulation revealed that stent implantation modified the haemodynamics by altering the shape of the blood vessel lumen, which decreased the Wall Shear Stress (WSS) and increased the Wall Shear Stress Gradient (WSSG) along the venous wall. Blood vessel shape (tapering and degree of deformation) also influenced how the haemodynamics in the stented blood vessel was impacted. In addition, the retrospective analysis on sheep blood vessels indicated that tissue thickness in the venous sinus could be correlated with the mean WSS patterns.

### Haemodynamic impact determines venous neointimal growth

Our sheep model suggested that stent-based neural interfaces may have altered the mechanical environment of the blood vessel wall, promoting tissue growth over months of stent implantation. Data analysis showed a negative correlation between the shear stress and associated tissue thickness. This suggests that venous tissue growth may be determined by reduced shear stress and agrees with findings in coronary stenting that link low shear stress to neointimal overgrowth^27^. Intimal growth has been observed in 14% of venous sinus stents (n = 473). The mechanism was uncertain but speculated to result from high shear stress and turbulence^28–30^. However, our study suggests that the superior sagittal sinus has a low-shear environment and is not tortuous enough to cause turbulence. Tissue growth in the superior sagittal sinus is more likely to be triggered by the reduction rather than elevation in shear stress. In addition, our work adds to the recent work of Opie et al.^14^ and John et al.^19^, who provided preliminary examinations of tissue growth patterns in sheep venous sinuses. A clearer understanding is needed of the complex pattern of tissue growth. Our computational models serve as additional case studies, leveraging existing data to explain these complex patterns using blood flow patterns. This approach addresses a key gap in previous studies and offers valuable insights for future stent-electrode design considerations.

With sheep-specific CFD models, we showed that the tissue thickness patterns were related to the shear stress pattern after stent implantation. By visualising the shear stress pattern, the CFD model has the potential to evaluate potential tissue growth in early-stage stent design testing and surgery planning for future stent-based neural interfaces. The novelty of our study lies in the complex tissue growth patterns observed, which are distinct from those seen in diseased arteries affected by atherosclerosis. Much of the results in the literature that rely on data from diseased arteries that are prone to plaque, thrombosis, and prolonged healing may not provide insights into normal tissue response in a healthy vein. Our study closely examined the tissue response from a healthy venous vessel to better understand the tissue growth patterns in our targeted veins.

Our study aimed to provide valuable insights, guidance, and simulation tools for future research in evaluating stent-electrode designs under normal venous flow conditions in healthy veins. To date, there are no studies showing tissue growth patterns and CFD of healthy veins. Unlike arterial walls, which often develop plaques consisting of fats, cholesterol, and aggregated platelets, the venous sinus walls do not have any pre-existing lesions or abnormal growth. The tissue growth patterns in venous sinuses are more varied and complex compared to the focal narrowing typically seen in arteries affected by atherosclerosis. Another key differences between work done previously on coronary arteries and our work in cerebral veins include the location and positions which also result in different boundary conditions. While CFD patterns may be somewhat similar due to the presence of stent struts, the ranges of Wall Shear Stress and Wall Shear Stress gradient on the venous wall were lower due to over-expansion and slow non-pulsatile blood flow conditions.

The retrospective data of the sheep model was prone to artefacts. Only three out of twelve sheep imaging datasets contained intact tissue thickness measurements. The streaking artefact from the metal electrodes mounted on the stent strut made there appear to be sudden changes in WSS and tissue thickness data. In future studies, better imaging techniques or image processing algorithms are needed.

In addition, the current CFD model only simulated haemodynamics right after implantation. Vascular remodelling is a complex and time-evolving process. An interplay could exist between haemodynamics and tissue growth. After reendothelialisation occurs, the neointimal tissue will merge over the stent surface. Haemodynamics could be further influenced by this growth, which would influence the subsequent tissue growth^31^. Intermittent measurement would be necessary to better understand the impact of stent haemodynamics over time. More work is required to validate the model and adapt it to human cerebral blood vessels which can provide significant benefit to clinicians and manufacturers in improving the design of the devices to suit patients.

### An oversized stent has more impact on venous haemodynamics

In previous works, most of the modelling studies use an idealised model without considering tapering or deformation due to the high computational cost^32,33^. Instead, a non-deformed model is commonly applied to study the haemodynamics of various stent designs and stented blood vessels with complex geometries^34–36^. While results from the non-deformed model provided some explanation of what is happening in the blood vessel, it grossly underestimated the haemodynamic impact of the intervention due to the inaccurate boundary conditions (i.e., blood vessel shape). Our results show that the inclusion of venous wall deformation resulted in a substantial difference in WSS and WSSG patterns, increasing the area subject to low WSS and high WSSG by 15% and 46%, respectively (Figs. 3, 4).

Our simulation results agreed with coronary artery stenting CFD studies, which showed deformation strongly affects the haemodynamics of various stent designs^37,38^. However, we showed greater and more non-uniform deformation along the venous sinus wall, which has not been quantitatively analysed in the literature. We found that a larger deformation caused a much greater elevation in the area subject to low WSS (Cases 2 and 3). Our findings highlight key differences between arteries and veins. On one hand, arteries, due to their greater stiffness, experience minimal lumen deformation and are highly influenced by the presence of stent struts^37^. On the other hand, veins, being more compliant, undergo significant lumen deformation and are less impacted by the presence of the thin stent struts of the venous sinus stent.

Our findings are crucial for stent size selection^39,40^ as suboptimal stent expansion leads to adverse clinical events^39^. In the literature, oversized stents with a ratio from 1.1 to 1.2 are commonly used to fully expand the diseased blood vessel without incomplete stent apposition and device migration^40^. However, our simulations suggest that using an oversized venous stent on a healthy venous sinus will substantially deform the venous wall unevenly and modify the blood flow patterns, promoting complex tissue growth patterns in the venous sinus. Considering the relationship between the area of low WSS and stent-to-vein ratio, further study will be required to determine the optimal oversizing parameter for the venous sinus stent. The deformed model will be the essential tool to determine the optimal venous stent designs, such as a tapered or a personalised stent that fits better in the venous sinus to mitigate the risks of venous tissue overgrowth. The presented venous model provides a better estimation of the haemodynamic effect and wall deformation in the venous sinus than previous models, thereby improving the testing of future venous implant designs and evaluation of venous neointimal growth.

### Blood vessel tapering challenges the optimal stent design

Our findings indicate that the commonly used oversizing stent may lead to increased tissue growth, particularly in tapered blood vessels. We observed that a larger deployment ratio at the narrower end of the original blood vessel stimulated more tissue growth. Future research should focus on developing tapered or customized stents that can reduce deformation while preventing migration. This study area can drive advancements in stent design, enhancing their effectiveness in clinical applications. Blood vessel tapering could make the selection of appropriate stent size more difficult by adding complexity to the blood flow pattern. Tapering is a common feature in the human venous sinus. For example, the human central sulcal vein has a mean distal (blood flow inlet) diameter of 2.3 mm and a mean proximal (outlet) diameter of 4.9 mm (tapering ratio b > 2) along 100 mm length^18^. The superior sagittal sinus naturally tapers along its entire length, with a ratio greater than 2 along 100 mm^18^. In the stented region, the inlet is approximately 10–20% narrower than the outlet, resulting in a tapering ratio of 1.1–1.2^18^. Post-stent implantation, the blood vessel wall was stretched by the self-expanded oversized stent to almost the same diameter as the stent itself (Fig. 2f), causing more pronounced deformation at the inlet and slight deformation at the outlet. This is reflected in Case 4.

However, in Case 5, a larger tapering ratio led to suboptimal stent expansion at the distal end, resulting in a large area of low WSS region at the distal end (Fig. 3). At the proximal end of the stented region, the narrowed inlet will suffer from a larger blood vessel wall deformation. At the distal end, the wider outlet may be prone to stent struts overhangs (malapposition). Poor stent apposition to the blood vessel wall is common and has been extensively studied due to its strong association with delayed stent incorporation and late thrombosis^41^. Previous CFD models have focused on malapposition in curvature^42^ and elliptical lumen shape^32^. Malapposition in tapered arteries has gained interest only in recent years^43^. Our simulation considered tapering in a deformed blood vessel model, which has not been studied before. Our results suggest that tapering needs to be considered when choosing the stent design, especially for venous sinus stents. It is essential to implant the stent with an appropriate expansion ratio that matches the blood vessel tapering ratio or even consider a customised stent with a tapered design that may fit better for an individual patient to mitigate the risks of adverse events. The presented CFD model would be a reliable platform to help determine stent designs for patients with tapered blood vessels.

### Limitations and future works

The CFD model had several assumptions. First, the mechanical simulations assumed that the venous wall was isotropic and elastic instead of anisotropic and hyper-elastic or viscoelastic. This is because relevant material data is unavailable in the literature. However, the mechanical model was used to generate a reasonable estimation of the blood vessel shape similar to the geometry from the reconstructed micro-CT images (Fig. 4). Mechanical simulation created the geometric details of how the stent was embedded in the venous wall, which could be used for flow simulation. It is necessary to note that solving the contact problem between the stent and vessel wall is computationally expensive^44^. A deformed model with more realistic material properties is recommended to yield a more realistic haemodynamic simulation. Given that the previous experiment had been completed, gathering additional animal-specific boundary conditions and material properties was not feasible. However, we are actively working on obtaining more accurate animal-specific or patient-specific input information that is currently lacking in the literature. This will allow us to achieve a more accurate and quantitative understanding of the events in the venous sinuses and determine optimal design parameters for future venous implants. In future work, we hope to validate the blood flow pattern and the material deformation of venous sinus wall in our models using animal experiments. The model will hold promise into efficient testing of optimal stent designs that minimize such deformation.

Second, the CFD boundary condition was taken from the average venous sinus blood flow in the literature. Assuming a constant flow rate may lose information relevant to neointimal tissue growth, such as instantaneous wall shear stress and its oscillatory behaviour within one cardiac cycle^45,46^. However, venous flow is relatively slower and less pulsatile than arterial flow. Implementing average blood flow to study venous haemodynamics is justified in the literature^28^. Future work will require a time-dependent velocity waveform if it is a patient-specific CFD model.

### A new frontier in endovascular neural interfaces

Currently, stent technology has been utilized beyond its traditional purposes, such as the SMART stent (stent with flow or pressure sensors)^47,48^ and stents with recording electrodes^49^. By adding sensors and electrodes, stents are empowered with more functions to serve as potential new or better treatment options for intractable diseases. Specifically, the endovascular neural interface has adapted to stent technology well, making high-fidelity brain signal recording minimally invasive^14,18^. Despite tissue proliferation in the blood vessel, the endovascular approach has great potential to improve outcomes for patients since it possesses a much lower risk of complication compared to conventional cortical electrodes^15^. The use of neurovascular stents and endovascular neural interfaces will continue to sprout over the next decade. Hence, it remains essential that the risks of stent complications are mitigated. There is a need to better characterize their impact on blood flow dynamics. This is especially important in blood vessels with complex geometry due to curvature and tapering, which increases the risk of stent size mismatch. CFD modelling enables a cost-efficient approach to examining the impact of different stent designs on blood vessels; it will provide critical insights that will enable the ongoing development, testing, and regulation of neurovascular stents and endovascular neural interfaces. CFD should be extensively used to evaluate the suitability of any new stent designs that are intended for patient use.

The use of neurovascular stents and endovascular neural interfaces will continue to increase over the next decade and there is a need to better characterise their impacts on blood flow dynamics. While previous research has provided some insight into tissue response, researchers are yet to determine the major driving force of tissue response to shed light upon future stent-electrode design. More importantly, ethical approvals for stent-electrode devices heavily rely on animal data, particularly from studies involving sheep, to facilitate the transition to human trials. Our sheep models serve as additional case studies, leveraging existing data to explain these complex patterns using blood flow patterns and offers valuable insights for future stent-electrode design considerations. The biomechanical environments of human and sheep venous sinuses are similar, characterized by slow, non-pulsatile blood flow and compliant venous wall material properties. The large deformation simulated by the human model aligned with observations in sheep. Specifically, greater deformation at the inlet, caused by stent over-expansion, resulted in lower Wall Shear Stress, potentially explaining the tissue growth patterns observed in sheep. Despite the anatomical difference between sheep and human venous sinus, which may result in tissue response, the similarity in blood flow patterns and material interactions between the metallic stent and venous wall suggests that the results can be extrapolated. Our results provide the first examination of haemodynamic changes and their correlation with neointimal thickness in endovascular neural interfaces after implantation. Studying blood flow is especially important when the stented blood vessel becomes complex due to blood vessel deformation and tapering. These results provide critical insights that will enable the ongoing development, testing, and regulation of neurovascular stents and endovascular neural interfaces.

## Data Availability

The datasets generated and/or analysed during the current study are available from the corresponding author upon reasonable request.
